# TLR4 Inhibition Attenuated LPS‐Induced Proinflammatory Signaling and Cytokine Release in Mouse Hearts and Cardiomyocytes

**DOI:** 10.1002/iid3.70133

**Published:** 2025-01-24

**Authors:** Christine W. Wiger, Trine Ranheim, Henriette Arnesen, Jarle Vaage, Søren E. Pischke, Arne Yndestad, Kåre‐Olav Stensløkken, May‐Kristin Torp

**Affiliations:** ^1^ Division of Physiology, Department of Molecular Medicine Institute of Basic Medical Sciences University of Oslo Oslo Norway; ^2^ Research Institute of Internal Medicine, Oslo University Hospital Oslo Norway; ^3^ Institute of Clinical Medicine University of Oslo Oslo Norway; ^4^ Department of Research and Innovation, Division of Emergencies and Critical Care Oslo University Hospital Oslo Norway; ^5^ Østfold Hospital Trust Grålum Norway

**Keywords:** DAMPs, heart, inflammation, LPS, PAMPs, sepsis

## Abstract

**Background:**

Sepsis is associated with myocardial injury and early mortality. The innate immune receptor Toll‐like receptor 4 (TLR4) can recognize pathogen‐associated‐molecular‐patterns (PAMPs) and damage‐associated molecular patterns (DAMPs); the latter are released during tissue injury. We hypothesized that TLR4 inhibition reduces proinflammatory signaling and cytokine release in: (1) LPS or *Escherichia coli*‐treated isolated mouse heart; (2) LPS‐treated mouse primary adult cardiomyocytes; and (3) the isolated heart during ischemia–reperfusion.

**Methods:**

Isolated C57BL/6N male mouse hearts were perfused for 120 min, with either LPS, *E. coli*, with and without CLI‐095 (TLR4 inhibitor). Primary adult mouse cardiomyocytes were treated with LPS or LPS + CLI‐095. Isolated hearts, exposed to 35 min of global ischemia, were treated with either vehicle or CLI‐095 during reperfusion. Infarct size was quantified by triphenyltetrazolium staining. Cytokine expression was analyzed with ELISA, western blot analysis, and qPCR.

**Results:**

In isolated hearts, *E. coli* increased the expression of proinflammatory cytokines (IL‐6 and CXCL2), which was not attenuated with TLR4 inhibition. TLR4 inhibition reduced expression (*p* = 0.004) and release of IL‐6 (*p* < 0.0001) in LPS‐exposed isolated hearts. LPS activated the nuclear‐factor κ‐light‐chain‐enhancer of activated B cells signaling pathway (NF‐κB) in primary adult cardiomyocytes. Moreover, TLR4 inhibition reduced LPS‐induced mRNA expression and release of IL‐6 in primary adult cardiomyocytes. Isolated hearts treated with CLI‐095 during reperfusion after ischemia (induced DAMPs release) showed reduced infarct size (39 ± 17% to 26 ± 8%, *p* = 0.034) and decreased IL‐6 release (*p* = 0.006).

**Conclusion:**

Inhibition of TLR4 reduced proinflammatory signaling and cytokine release in LPS‐treated and ischemia–reperfused isolated mouse hearts and in primary adult murine cardiomyocytes.

## Graphical abstract

Inhibiting TLR4 reduce LPS‐induced IL‐6 and CXCL2 release in perfused isolated mouse hearts (A, B). Inhibiting TLR4 at reperfusion reduce infarct size in perfused isolated mouse hearts after 30 minutes of ischemia (C). Inhibiting TLR4 at reperfusion reduce IL‐6 release in isolated mouse heart after myocardial infarction (D).

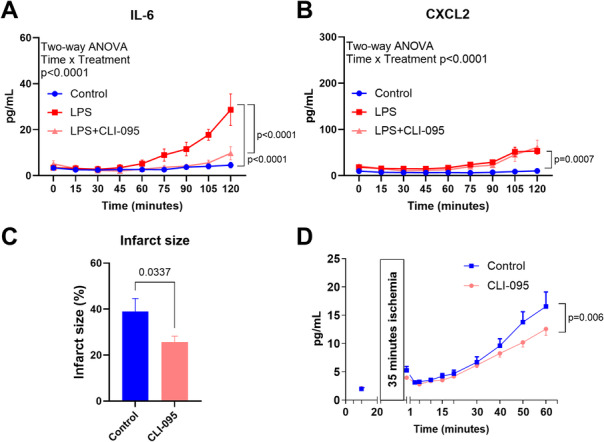

**Abbreviations**
BPM: beats per minute
CXCL2: chemokine (C‐X‐C motif) ligand 2
DAMPs: damage‐associated molecular patterns
DMSO: dimethyl sulfoxide
*E.coli*
: *Escherichia coli*
ERK: extracellular signal‐regulated kinase
HMGB1: high mobility group box 1 protein
IFNγ: interferon γ
IL‐18: interleukin‐18
IL‐1β: interleukin 1
IL‐6: interleukin‐6
IR: ischemia–reperfusion
IRI: ischemia–reperfusion injury
JNK: c‐Jun N‐terminal kinases
LDH: lactate dehydrogenase
LPS: lipopolysaccharide
LV: left ventricle
LVEDP: left ventricle end‐diastolic pressure
LVSP: left ventricle systolic pressure
MAPK: mitogen‐activated protein kinase pathways
MCP‐1: monocyte chemoattracttant protein‐1
mtDNA: mitochondrial DNA
MTT: 3‐[4,5‐dimethylthiazol‐2‐yl]‐2,5 diphenyl tetrazolium bromide
Mydd88: myeloid differentiation primary response 88
NF‐κB: nuclear factor κ‐light‐chain‐enhancer of activated B cells
PAMPs: pathogen‐associated molecular patterns
PRRs: pattern recognition receptors
TLR2: Toll‐like receptor 2
TLR4: Toll‐like receptor 4
TLR9: Toll‐like receptor 9
TLRs: Toll‐like receptors
TNF: tumor necrosis factor
TTC: 2,3,5‐triphenyltetrazolium chloride

## 1 Introduction

Sepsis is a systemic inflammatory response, defined as “a life‐threatening organ dysfunction caused by a dysregulated host response to infection” [1]. Even with advanced treatment and decreasing death rates, the global burden of the disease is still high, with 48.9 million cases of sepsis and 11 million sepsis‐related deaths worldwide [2]. During the course of sepsis, there is a high risk of developing cardiovascular complications such as myocardial infarction, heart failure, and stroke [3].

Bacteria have molecular patterns structurally different from the host, termed pathogen‐associated molecular patterns (PAMPs) [4]. Sepsis‐induced tissue injury can cause release of endogenous substances that exacerbate whole‐body inflammation. These molecules are termed damage‐associated molecular patterns (DAMPs) [5]. DAMPs and PAMPs are recognized by both immune cells and nonimmune cells via pattern recognition receptors (PRRs), located either on the plasma membrane, intracellularly in endosomes, or in the cytosol [6]. Toll‐like receptors (TLRs) belong to the PRR family and 13 TLRs have been identified [7]. TLR expression has been shown in cardiomyocytes and in cardiac fibroblasts [8] and the most abundant TLR in the mammalian heart is Toll‐like receptor 4 (TLR4) [9]. Consequently, cardiomyocytes and cardiac fibroblasts can respond to PAMPs and DAMPs in a similar way as immune cells [10].

One of the most common pathogen causing sepsis are Gram‐negative bacteria, such as *Escherichia coli* (*E. coli*) [11], which triggers whole‐body inflammation and can potentially lead to sepsis [12]. Common for all Gram‐negative bacteria is the lipopolysaccharide (LPS) located on the outer cell wall which is a potent agonist for TLR4 [13]. LPS injection in healthy young men was found to increase temperature, heart rate, respiratory rate, white blood cell count, and secretion of C‐reactive proteins, tumor necrosis factor (TNF), and interleukin‐6 (IL‐6), demonstrating a high inflammatory potential of LPS [14].

Ligand binding to TLR4 activates nuclear factor κ‐light‐chain‐enhancer of activated B cells (NF‐κB) and mitogen‐activated protein kinase pathways (MAPK). The result is release of inflammatory cytokines such as IL‐6 and chemokine (C‐X‐C motif) ligand 2 (CXCL2) [15, 16]. Murine cardiomyocytes have been shown to express both IL‐6 and CXCL2 [17]. TLR4 may play a crucial part in in vivo experiments of ischemia–reperfusion injury (IRI) and DAMP‐induced damage in the heart, emphasized by reports of decreased infarct size in mice lacking TLR4 [18]. Intraperitoneal injection of a TLR4‐specific inhibitor (CLI‐095), reduces LPS‐induced cardiac dysfunction, reduces TLR4 expression in the myocardium, and decreases serum levels of TNF [19]. Moreover, TLR4‐deficient mice have higher survival rate during lethal polymicrobial sepsis compared to mice with normal TLR4 expression [20]. Although in vivo experiments are instrumental, it is difficult to study and interpret the direct effect of PAMPs on the whole heart and in cardiomyocytes. Moreover, it is not known if TLR4 inhibition would reduce cardiac inflammation when the heart is exposed to a complex mixture of PAMP, mimicking sepsis. Our aim was therefore to expose the ex vivo perfused heart to a sepsis‐like state using sonicated *E. coli* fragments or LPS, with or without TLR4 inhibition. We hypothesized that inhibition of TLR4 would reduce inflammatory signaling and cytokine release. As we found that TLR4 inhibition only reduced inflammatory signaling after LPS stimulation, LPS was further studied in cardiomyocytes. Furthermore, we wanted to compare the inflammatory signaling from PAMP exposure with DAMP release. We used a known model of ischemia and reperfusion (IR) in the isolated heart and hypothesized that TLR4 inhibition would reduce post ischemia damage and immune response.

## 2 Materials and Methods

Reagents were purchased from Sigma‐Aldrich (St. Louis, MO, USA) unless otherwise stated.

### 2.1 Mice

Mouse experiments were approved and conducted in accordance with the Norwegian Food Safety Authority (FOTS ID 12211 and 18679) and the guidelines from Directive 2010/63/EU of the European Parliament on the protection of animals used for scientific purposes. C57BL/6N male wild‐type mice (Scanbur, Nittedal, Norway) weighing 25–30 g were kept in a controlled environment with 12:12 h light:dark period, 23°C, 55%–60% humidity, and free access to water and chow (RM3, Scanbur). The mice were acclimatized for a minimum of 7 days before any experiments. Anesthesia was induced by intraperitoneal injection of sodium pentobarbital (50 mg/kg, Norges Apotekerforening, Oslo, Norway). After heparinization (500 IU, Leo Pharma A/S, Denmark), the mice were euthanized by cervical dislocation.

### 2.2 Langendorff Heart Perfusion

Langendorff heart perfusion was performed as described previously [21]. After anesthesia, mouse hearts were quickly excised and cannulated through aorta and retrogradely perfused with Krebs‐Henseleit buffer (in mM: NaCl 118.5, NAHCO_3_ 25.0, KCl 4.7, KH_2_PO_4_ 1.2, MgSO_4_/7H_2_O 1.2, Glucose/1H_2_O, 11.1, CaCl_2_ 2.4). Perfusion pressure was constant at 70 mmHg set by either (1) gravity or (2) a peristaltic pump.

#### 2.2.1 Experimental Series I: LPS and *E. coli*

The LPS/*E. coli* experiment was performed with glass equipment that tolerate both high temperatures and ethanol. The reason for having a separate set‐up for the LPS/*E. coli* experiment was because LPS easily binds to plastic and could therefore potentially contaminate the different experimental groups. In this recirculating set‐up, hearts were stabilized by perfusion for 20 min before randomly included in the following groups with different treatment (*n* = 6): (i) no treatment, (ii) 1 × 10^7^ *E. coli*, (iii) 1 × 10^7^ *E. coli* + 1 µM CLI‐095 (Invivogen, San Diego, California), (iv) 1 µg/mL LPS, or (v) 1 µg/mL LPS + 1 µM CLI‐095. *E. coli* was strain LE392 (ATCC, VA, USA) and used as previously described [22]. Hearts were perfused for 120 min and perfusate (1 mL) was collected at 0, 15, 30, 45, 60, 75 90, 105, and 120 min in perfusion. The perfusate and the perfused hearts were snap frozen in liquid nitrogen and stored at −80°C for further analyses.

#### 2.2.2 Experimental Series II: IR

The experimental set‐up for IR was as follows: 20 min stabilization, 35 min global ischemia, and 60 min reperfusion. A handmade, fluid‐filled, plastic balloon introduced into the left ventricle (LV) via the left atrium measured left ventricular pressures. LV end‐diastolic pressure (LVEDP) was adjusted to 5–10 mmHg during stabilization. Data acquisition was by Powerlab software, Labchart (ADInstruments Ltd., Oxford, UK). Hearts that exceeded the following criteria were excluded from the study: Aortic cannulation time > 3 min, coronary flow < 1 and > 4 mL/min, LV systolic pressure (LVSP) < 60 mmHg, heart rate < 220 beats per minutes (bpm). After start of perfusion, the hearts were stabilized for 20 min, followed by 35 min of global ischemia and 60 min of reperfusion. The hearts were randomized into two groups. (1) The hearts (*n* = 11) were from start of reperfusion treated with 1 µM CLI‐095; (2) Controls (*n* = 9) were perfused with the vehicle, 0.04% dimethyl sulfoxide (DMSO). After reperfusion, hearts were sectioned in 1 mm thick slices and stained with 1% triphenyltetrazolium chloride for blinded infarct size quantification using Adobe Photoshop version 13.0. Coronary perfusates were collected for 1 min after 10 min of stabilization and after reperfusion for 1, 3, 5, 10, 15, 20, 30, 40, 50, and 60 min. Perfusate and heart tissue were snap frozen in liquid nitrogen and stored at −80°C until further analyses. Perfusates were analyzed for lactate dehydrogenase (LDH) using a Cytotoxicity Detection Kit (Roche, Penzberg, Germany) according to the manufacturer's protocol.

### 2.3 Primary Adult Mouse Cardiomyocytes

#### 2.3.1 Isolation Procedure

Primary adult cardiomyocytes were isolated according to the procedure reported by O'Connell et al. [23]. Hearts were retrogradely perfused (perfusion buffer in mM: NaCl 120.4, KCl 14.7, KH_2_PO_4_ 0.6, Na_2_PO_4_ 0.6, MgSO_4_ 1.2, Na‐HEPES liquid 10.0, glucose 5.5, NAHCO_3_ 4.6, taurine 30.0, BDM (2,3‐butanedione monoxime) 10). Initially, heart perfusion was performed with perfusion buffer alone, then with addition of 1.3 mg/mL Collagenase type 2 (#4177, batch 45D15719, activity 355 U/mg, Worthington Biochemical, Lakewood, NJ, USA). After collagenase inhibition with HyClone Bovine Calf Serum (FBS, #SH30073.03, GE Healthcare Life Sciences, Marlborough, MA, USA) diluted in perfusion buffer (1:10), cells were separated by gently pipetting and centrifugation at 20*g* to separate cardiomyocytes from non‐cardiomyocytes. Centrifugations at 20*g* were repeated in total four times with increasing concentration of Ca^2+^ (in µM: 12.5, 100, 400, and 900) to purify the cardiomyocyte isolate. Cardiomyocytes were resuspended in plating medium (MEM with Hanks BSS (#M5775) supplemented with 10% FBS, 100 U/mL penicillin‐streptomycin, 2 mM l‐glutamine, and 10 mM BDM) and plated on laminin‐coated plates (1 µg/cm^2^, #354232, Corning, NY). After 1–2 h, plating medium was replaced by short term medium (MEM with Hanks BSS supplemented 100 U/mL penicillin‐streptomycin, 2 mM l‐glutamine, 0.1% BSA (low endotoxin, fatty acid free), and 1 mM BDM). Any treatment was mixed in short term medium before added to the cells. Cells were incubated in air with 2% CO_2_ at 37°C.

#### 2.3.2 In Vitro Imaging of NF‐κB Activity to Demonstrate TLR4 Signaling in Cardiomyocytes

Cardiomyocytes were isolated as described above from NF‐κB firefly luciferase reporter mice and treated with 10 ng/mL LPS or vehicle control [24]. Luciferase activity was assessed with an IVIS 100 imaging system and exposure time was set to 5 min.

#### 2.3.3 Viability Assay

To evaluate viability of cardiomyocytes, cells were cultured in Lab‐Tek II eight well glass chamber slides (Thermo Fisher Scientific Inc.), and treated according to protocol. The treated cardiomyocytes were stained with 1 µM Hoechst 33258 (Thermo Fisher Scientific Inc.), 16.6 nM Mitotracker Deep Red FM (Thermo Fisher Scientific Inc.), and 12.5 µM Propidium iodide solution for 30 min at 37°C, 2% CO_2_. After incubation, cells were washed twice in PBS and fixed for 10 min at 37°C, 2% CO_2_ with 2% paraformaldehyde in PBS. Pre‐heated gelatin/glycerol‐based mounting medium was added before the slides were sealed with a cover glass. The cells were analyzed with an Olympus Scan^R High‐Throughput microscope with corresponding acquisition and analysis software. The slides were scanned at ×10 magnification and with equal automatic settings. Images were taken from 25 fixed positions in each well. Hoechst staining was used in an object based autofocus setting. Mitotracker staining was used to count the total number of cells analyzed, whereas propidium iodide was used as an indicator of cell death as this marker do not penetrate viable and intact cell membranes. Viability results are either presented as a ratio of total number of propidium iodide stained nuclei and total number of nuclei, or as elongation factor (ratio between length and width of the cardiomyocyte).

In addition, the colorimetric assay, MTT (3‐[4,5‐dimethylthiazol‐2‐yl]‐2,5 diphenyl tetrazolium bromide) was used according to the manufacturer's protocol, to further evaluate cardiomyocyte viability.

### 2.4 ELISA

ELISA DuoSets were purchased from R&D systems (IL‐6: DY406‐05 and CXCL2: DY452‐05, R&D Systems Inc., Minneapolis, MN) and the protocol was conducted according to the manufacturer. In short, capture antibodies were diluted in PBS and added to high‐binding 96‐well (Corning) plates 1 day in advance of the experiment. Plates were blocked with reagent diluent (1% BSA in PBS) and washed with washing buffer (0.05% Tween 20 in PBS). Substrate solution was purchased from Life Technologies (TMB Single solution, Thermo Fisher Scientific Inc.), and H_2_SO_4_ was used as stop solution. Absorbance was measured at 450 and 570 nm with BioTek PowerWave XS (BioTek Instruments Inc.). A standard curve on each plate was used to quantify cytokine release.

### 2.5 Western Blot Analysis

Heart tissue were crushed in a mortar with liquid nitrogen and 50 mg tissue was lysed in RIPA buffer (1:10 RIPA and 1:100 Halt Protease and Phosphatase Inhibitor Cocktail (Thermo Fischer Scientific Inc.) diluted in MQ water). Protein samples were homogenized with a tissue homogenizer (Benchmark Scientific D1000) and centrifuged at 10,000*g* for 7 min. For complete denaturation of proteins, 1% SDS was added to each sample and left in RT for 15 min with occasional vortexing. Protein concentration were measured with Micro BCA Protein Assay Kit (Thermo Fischer Scientific Inc.) according to manufactures instructions. Protein extracts were mixed with 6X Laemmli buffer (60 mM Tris‐HCl, pH 6.8, 47% glycerol, 12% SDS, 0.06% Bromophenol Blue, and 600 mM dithiothreitol [DTT]) and heated at 98°C for 5 min. Protein extracts were loaded (15 µg) and separated on a 4%–20% Criterion Pre‐Cast Gel (BioRad, Hercules, CA) at 200 V in Tris/glycine/SDS buffer at room temperature. Proteins were transferred to a nitrocellulose membrane (BioRad) at 100 V in ice‐cold Tris/glycine/methanol buffer. Total loaded proteins were stained with 0.1% Ponceau solution (Merck‐Millipore Massachusetts, USA). Membranes were blocked in 5% nonfat dry milk (BioRad) for 1 h in room temperature, before primary antibody labeling at +4°C overnight. Primary antibodies were detected with HRP‐conjugated secondary antibodies for 1 h in room temperature. For visualization, membranes were incubated with SuperSignal West Dura Extended Duration Substrate (Thermo Fisher Scientific Inc.), and developed with ChemiDoc Touch Imaging System (BioRad). Band intensity was analyzed with Image Lab Software (BioRad). Antibody manufacturer and catalog numbers are listed in (Supporting Information S1: Table 1).

### 2.6 mRNA Isolation and qPCR

Total RNA was isolated with RNeasy Mini kit (Qiagen, Hilden, Germany) according to the manufacturer's protocol. In short, RNA isolate was treated with DNase (Qiagen) before eluted in RNase‐free water. Total RNA concentration was measured with Nano Drop 1000 Spectrophotometer (Thermo Fisher Scientific Inc.). cDNA of the mRNA was made with qScript cDNA Synthesis Kit (Quanta Biosciences, Chicago, USA) according to the manufacturer's protocol and synthesized in a T3 Thermocycler (Biometra, Gottingen, Germany). mRNA expression was measured with Power SYBR Green PCR Master Mix (Thermo Fisher Scientific Inc.), and qPCR 7900HT Fast Real‐Time PCR system SDS2.3 (Thermo Fisher Scientific Inc.). Primer sequences are shown in Supporting Information S1: Table 2.

### 2.7 Statistical Analysis

All statistical analyses were performed with GraphPad Prism version 8 Software (GraphPad Software Inc., San Diego, CA). Data sets were analyzed with either two‐way *t* test, mixed effects analysis, one‐way or two‐way ANOVA with Bonferroni, Šidák's or Dunn's multiple comparisons test. Langendorff data were analyzed with repeated measures two‐way ANOVA and Bonferroni's or Tukeys multiple comparison test.

## 3 Results

### 3.1 LPS Triggered TLR4‐Dependent IL‐6 Release and Inflammatory Signaling in Isolated Perfused Hearts

**Figure 1:**
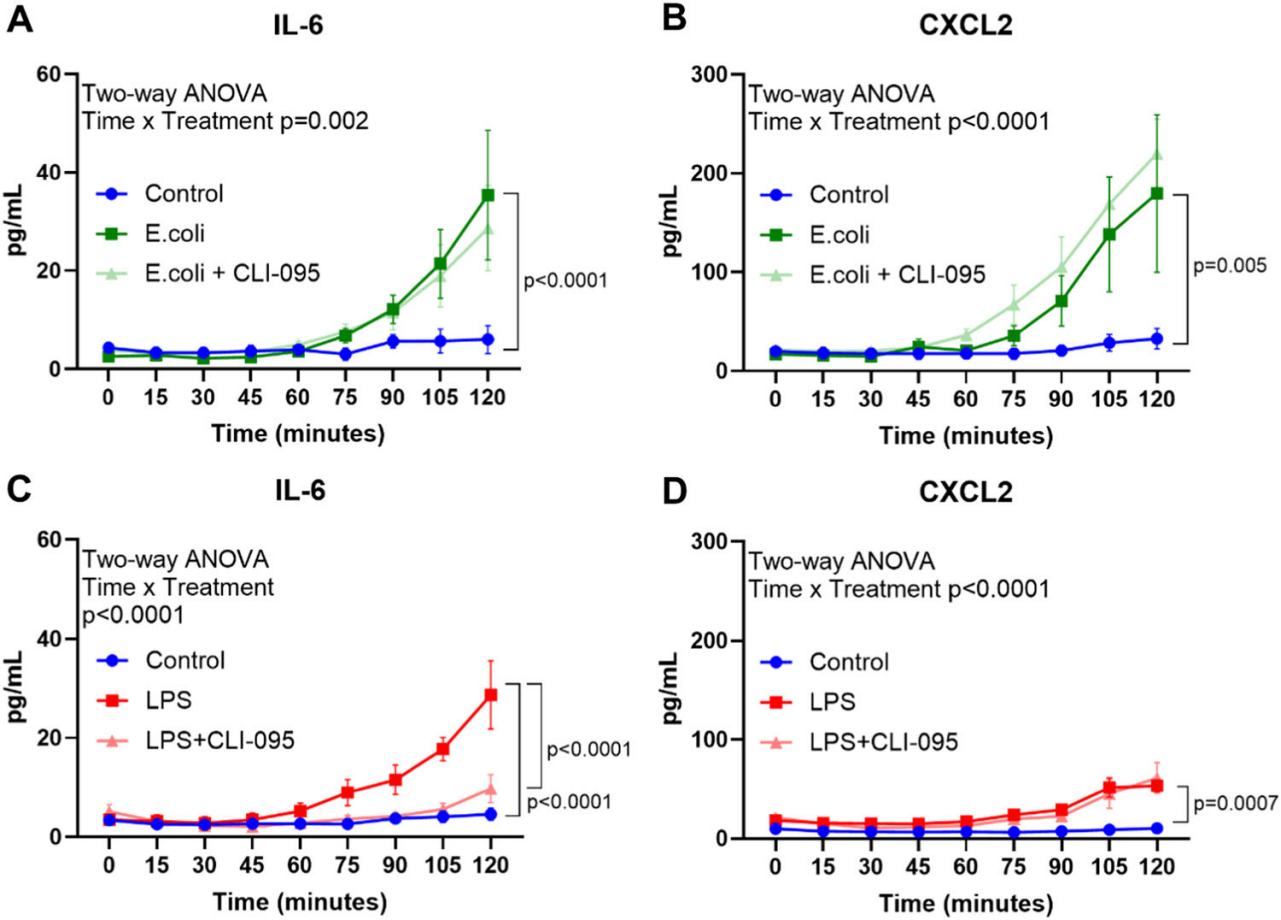
PAMPs induced release of IL‐6 and CXCL2. Isolated, buffer‐perfused hearts (*n* = 5–6) were introduced to either (i) no treatment (NT; blue), (ii) *Escherichia coli* (*E. coli*; dark green), (iii) *E. coli* and CLI‐095 (*E. coli* + CLI; light green), (iv) lipopolysaccharide (LPS; red), or (v) LPS and CLI‐095 (LPS + CLI; light red) for 120 min. Cytokine release measured in coronary perfusates collected at 0, 15, 30, 45, 60, 75, 90, 105, and 120 min for interleukin 6 (IL‐6, A, C) and C‐X‐C Motif Chemokine Ligand 2 (CXL2, B, D). Data are presented as mean ± SEM and analyzed by two‐way ANOVA with Tukey's multiple comparisons.

**Figure 2:**
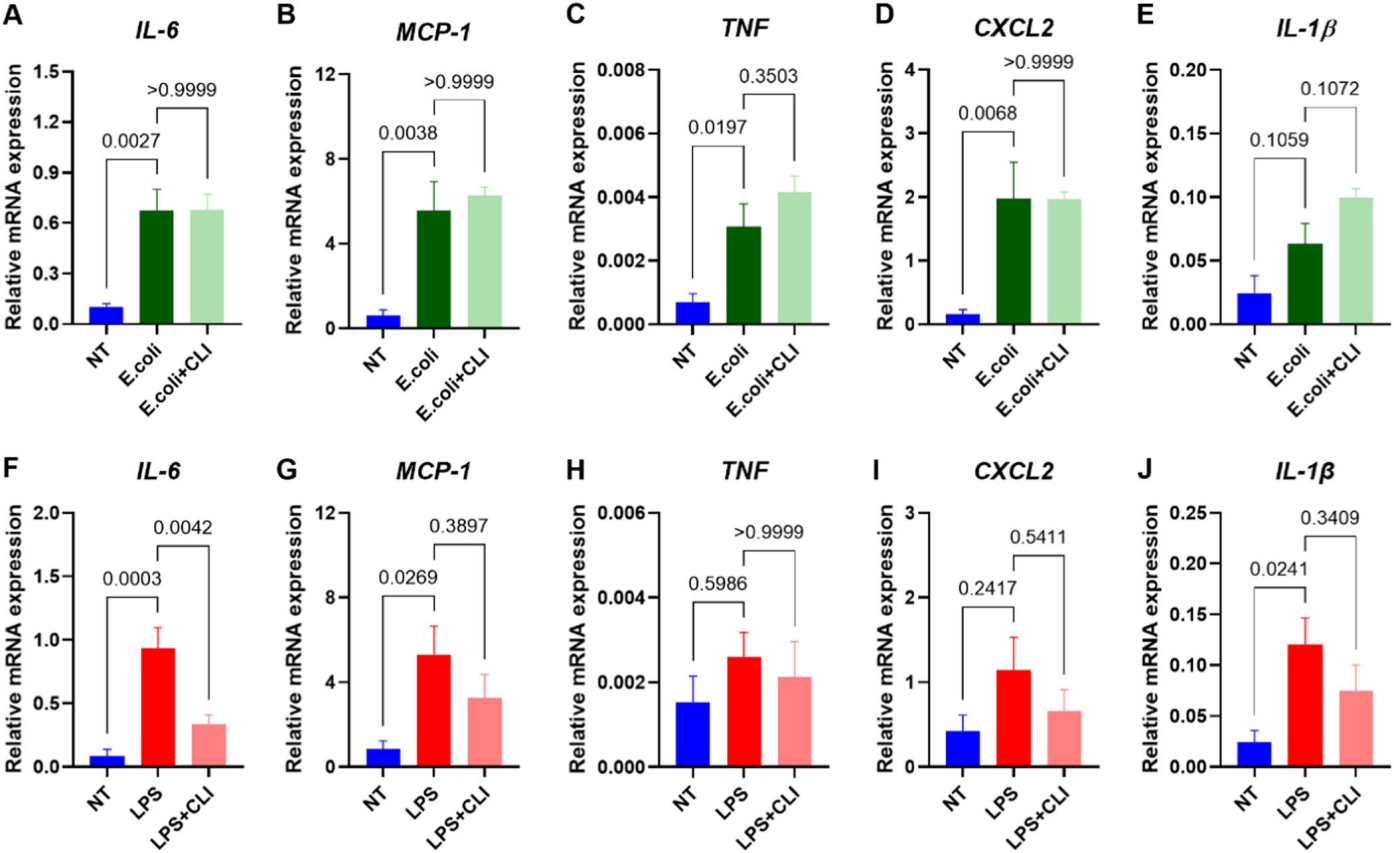
PAMPs activated proinflammatory signaling in the isolated perfused heart. Isolated, buffer‐perfused hearts (*n* = 5–6) were introduced to either (i) no treatment (NT; blue), (ii) *Escherichia coli* (*E. coli*; dark green), (iii) *E. coli* and CLI‐095 (*E. coli* + CLI; light green), (iv) lipopolysaccharide (LPS; red), or (v) LPS and CLI‐095 (LPS + CLI; light red) for 120 min. Relative mRNA expression of proinflammatory cytokines quantified in tissue RNA with qPCR. Data are presented as mean ± SEM and analyzed with one‐way ANOVA and Bonferroni's multiple comparisons. CXCL2, C‐X‐C motif chemokine ligand 2; IL‐1β, interleukin 1β; IL‐6, interleukin 6; MCP‐1, monocyte chemoattractant protein‐1; TNF, tumor necrosis factor.

Isolated and buffer perfused mouse hearts were exposed to either *E. coli* or LPS with and without the TLR4 inhibitor, CLI‐095. Upstream (NF‐κB and MAP kinase pathway), and downstream activation (cytokine expression), and cytokine release were quantified. Both *E. coli* and LPS exposure increased IL‐6 and CXL2 release, but inhibiting TLR4 only reduced LPS induced IL‐6 release (Figure 1). We found no significant changes in upstream signaling in hearts exposed to *E. coli*, while LPS significantly triggered NF‐κB signaling (Supporting Information S1: Figure 1A). Both *E. coli* and LPS increased cytokine mRNA expression of *IL‐6*, and monocyte chemoattractant protein‐1 (*MCP‐1*) (Figure 2A,B,F,G). In addition, *E. coli* perfusion increased expression of *TNF* and *CXCL2* (Figure 2C,D), whereas LPS increased expression of interleukin 1β (*IL‐1β*) (Figure 2J). Interleukin‐18 (*IL‐18*) and *interferon γ (IFNγ*) expression remained unchanged in all groups (Supporting Information S1: Figure 1).

### 3.2 LPS Activated the NF‐κB Signaling Pathway to Express and Release Proinflammatory Cytokines in Primary Adult Cardiomyocytes

**Figure 3:**
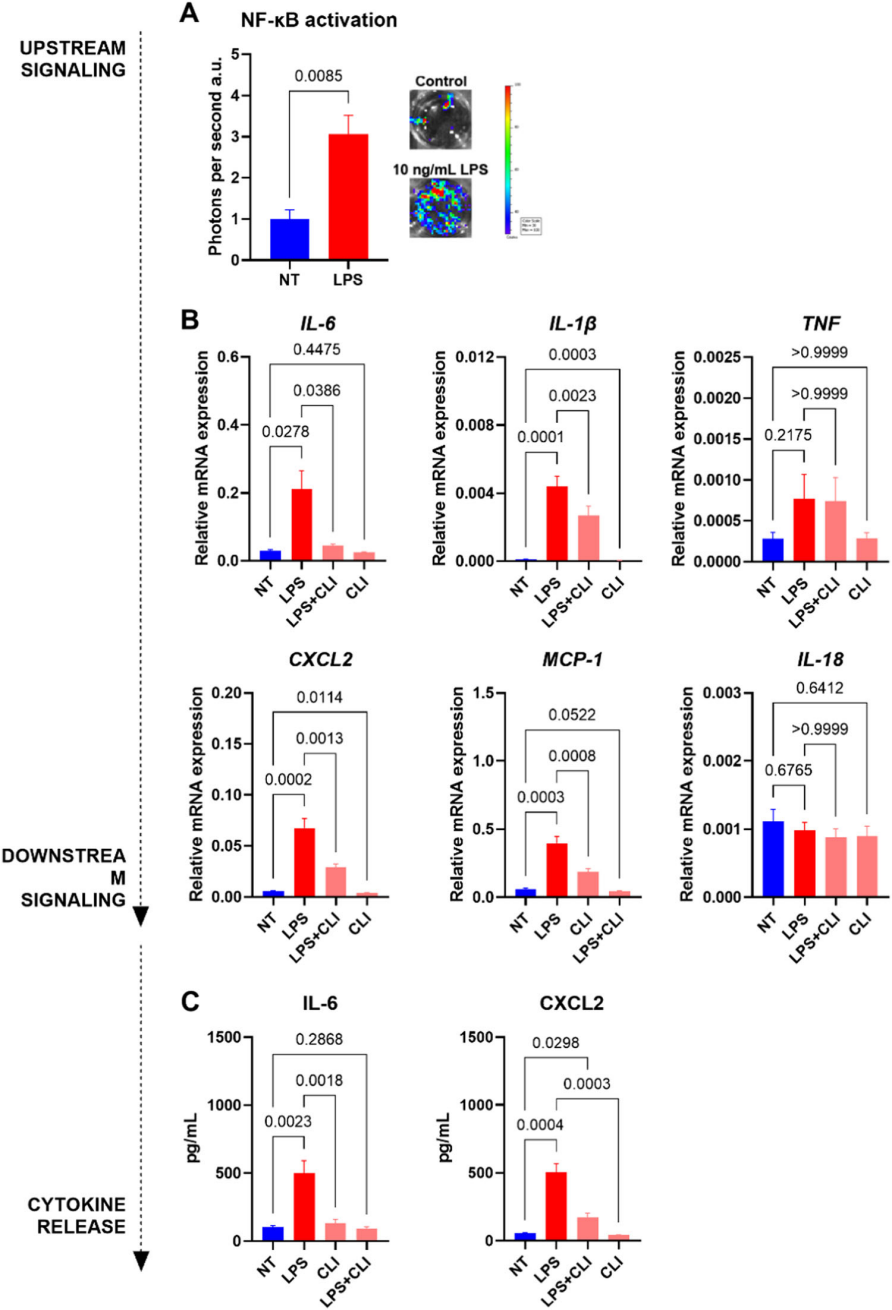
Proinflammatory response in LPS treated primary adult murine cardiomyocytes. Mice (*n*= 9‐10) cardiomyocytes were exposed to (i) no treatment (NT; blue), (ii) lipopolysaccharide (LPS; red), (iii) LPS and CLI‐095 (LPS + CLI; light red), or (iv) CLI‐095 (CLI; light pink). (A) Intracellular upstream NF‐κB activation in cardiomyocytes isolated from NF‐κB luciferase reporter mice (*n* = 6). (B) Relative mRNA expression of proinflammatory cytokines quantified with qPCR (*n* = 9–10). (C) Cytokine release measured in cell culture media with ELISA. Data are presented as mean ± SEM and analyzed with two‐tailed *t* test (A), one‐way ANOVA (B; IL‐6, IL‐1β, TNF, CXCL2) or mixed‐effects model (B; MCP‐1 and IL‐18) and Bonferroni's multiple comparisons (B, C). CXCL2, C‐X‐C motif chemokine ligand 2; IL‐18, interleukin 18; IL‐1β, interleukin 1β; IL‐6, interleukin 6; MCP‐1, monocyte chemoattractant protein‐1; NF‐κB, Nuclear factor κB; TNF, tumor necrosis factor.

To investigate the LPS‐triggered inflammatory signaling in primary adult cardiomyocytes, cardiomyocytes were isolated from NF‐κB luciferase reporter mice. Exposure to LPS increased NF‐κB activation twofold in adult cardiomyocytes (Figure 3A). Moreover, cardiomyocytes treated with LPS increased mRNA expression of *IL‐6*, *IL‐1β*, *CXCL2*, and *MCP‐1*, all of which were reduced with TLR4 inhibitor (Figure 3B). *TNF* and *IL‐18* expression was not altered by LPS. Furthermore, IL‐6 and CXCL2 increased in the cell culture media after LPS exposure which was reduced after TLR4 inhibition (Figure 3C).

### 3.3 Cardiomyocyte Viability and Macroscopic Morphology Were Not Altered by LPS

As an excessive inflammatory cytokine production can lead to cell death and tissue damage, we wanted to investigate if inflammation triggered by LPS could be harmful for the cardiomyocytes. However, we found no evidence that supports a change in cardiomyocyte viability (Supporting Information S1: Figure 2A) and MTT (Supporting Information S1: Figure 2B), nor any change in cell morphology (Supporting Information S1: Figure 2C) for the doses of LPS used in the study.

### 3.4 DAMPs Released in Ischemia‐Reperfusion Injury Trigger Inflammation Through TLR4 and Exacerbate Cardiac Injury

**Figure 4:**
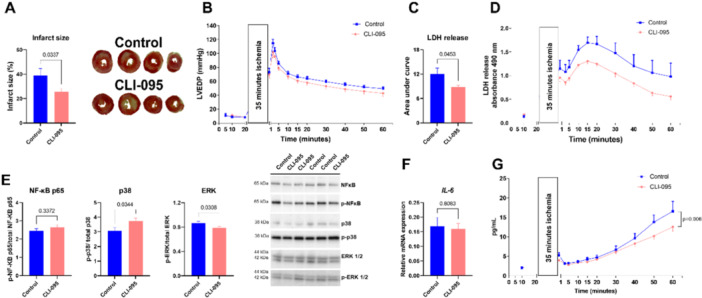
DAMPs released in ischemia‐reperfusion injury trigger toll‐like receptor 4 and exacerbate cardiac injury**.** Isolated, buffer‐perfused mouse hearts were exposed to 35 min ischemia followed by 60 min reperfusion. In reperfusion, hearts were treated either with a vehicle (control [0.04% DMSO]; blue) or CLI‐095 (CLI, light pink). (A) Infarct size measured in TTC stained heart slices (*n* = 9–11), (B) LVEDP measured isovolumetrically with a plastic balloon in the left ventricle (*n* = 6–9), (C) LDH release in coronary perfusate presented as the area under the curve, and (D) throughout the experimental protocol (*n* = 9–11), (E) western blot analysis of upstream proinflammatory signaling proteins (*n* = 9–11), (F) mRNA expression of *IL‐6* quantified with qPCR (*n* = 9–11), and (G) IL‐6 released into coronary perfusate (*n* = 8–11). Data are presented as mean ± SEM and analyzed with two‐tailed *t* test (A, C, E, F) or two‐way ANOVA (B, D, and G) and Bonferroni's multiple comparisons. DMSO, dimethyl sulfoxide; ERK, extracellular signal‐regulated kinase; IL‐6, interleukin 6; LDH, lactate dehydrogenase; NF‐κB, nuclear factor κB; TTC, 2,3,5‐triphenyltetrazolium chloride.

It is not known if DAMPs released from damaged cells activate TLR4 in cardiac cells. Therefore, isolated hearts were subjected to IRI with or without CLI‐095 during reperfusion in a Langendorff set‐up that excludes circulating immune cells (Figure 4). Blocking TLR4 with CLI‐095 significantly reduced infarct size compared to control (Figure 4A). TLR4 inhibition reduced LVEDP, but this was not significant (Figure 4B). Release of LDH, another indicator of cellular injury, was lower in CLI‐095 treated hearts (Figures 4C and 4D). Upstream inflammatory signaling of NF‐κB showed increased p38 activation and decreased ERK activation after CLI‐095 treatment when tissue was sampled after 60 min of reperfusion (Figure 3E). However, total NF‐κB activation was unaltered (Figure 3E). A similar trend was observed for the *IL‐6* cytokine with unaltered expression in tissue after 60 min of reperfusion (Figure 3F). Interestingly, IL‐6 released into the coronary perfusate was reduced in CLI‐095 treated hearts compared to control during reperfusion (Figure 3G). Furthermore, cardiac cellular debris and LPS activated NF‐κB via TLR4 in HEK cells overexpressing TLR4 (Supporting Information S1: Figure 4). Although LPS increased mRNA expression of *IL‐6, CXCL2, IL‐1β*, and *MCP‐1* in cardiomyocytes, only *CXCL2* was significant (Supporting Information S1: Figure 3A–D). After hypoxia‐reoxygenation the LPS induced increase in mRNA expression was significant for *IL‐6, IL‐1β*, and *MCP‐1* (Supporting Information S1: Figure 3A–D).

## 4 Discussion

The present study demonstrates that *E. coli* and LPS increased myocardial cytokine expression and release. However, inhibiting TLR4 only reduced LPS‐induced inflammatory activation. LPS activated NF‐κB in primary mouse cardiomyocytes and IL‐6 expression and release in ex vivo perfused mouse hearts were reduced by TLR4 inhibition. TLR4 inhibition also reduced infarct size and IL‐6 release in a model that induced DAMP release from isolated, perfused mouse hearts. In parallel, whole heart debris increased NF‐κB activity in reporter cells, which was reduced when inhibiting the TLR4 receptor. Taken together, our findings indicate that LPS activate an acute immune response in cardiomyocytes, which can be reduced by blocking TLR4. The immune response with our doses of LPS is comparable with the effect of cellular DAMPS after myocardial infarction.

### 4.1 *E. coli* and TLR4 inhibition in isolated hearts

Perfusion of isolated mouse hearts with *E. coli* increased downstream cytokine mRNA expression and release of IL‐6 and CXCL2. Blocking TLR4 had no effects on this release. Neither NF‐κB nor the investigated MAP‐kinases were activated after 120 min of perfusion. However, 120 min may be too long after the first exposure to be able to capture full activation of MAP‐kinases. Activation of NF‐κB and MAP‐kinases are dynamic and often seen early after exposure [25].

In the two most common models of experimental sepsis, intraperitoneal/intravenously administration of *E. coli* or cecal ligation and puncture, proinflammatory cytokine production increases [17, 26]. In mice, *E. coli* causes inflammation and oxidative stress [27]. However, its effect in different organs can vary [27]. Our study showed increased cytokine release in the isolated heart after *E. coli* and LPS exposure. We did not see a decreased cytokine release after TLR4 inhibition in isolated perfused hearts with *E. coli*. This suggests that the proinflammatory signaling induced by *E. coli* is not only TLR4 dependent in the heart.

*E. coli* contains a variety of PAMPs that can activate several PRRs [28] such as Toll‐like receptor 2 (TLR2) [28] and Toll‐like receptor‐9 (TLR9) [29]. TLR2 primarily recognize bacterial lipoproteins and peptidoglycans, both of which are components of *E. coli* [28]. TLR9 is primarily known for recognizing unmethylated CpG motifs in bacterial DNA [29]. Because many PRRs can be activated during sepsis, blocking only TLR4 is not sufficient to reduce cytokine induction.

### 4.2 LPS and TLR4 Inhibition in Isolated Heart and Isolated Cardiomyocytes

Perfusing isolated mouse hearts with LPS increased mRNA expression of *IL‐6*, *IL‐1β*, and *MCP‐1*. However, only *IL‐6* mRNA expression and IL‐6 protein release decreased when inhibiting TLR4. In heart tissue, we could not differentiate in which cardiac cell type expression and release originated. While cardiac fibroblasts are known to produce IL‐6 [30], cardiomyocytes can also produce IL‐6 in response to injury [31]. We found that LPS increased *IL‐6* and *CXCL2* expression and release in isolated cardiomyocytes, which was reduced by TLR4 inhibition. Moreover, LPS increased NF‐kB activation in primary cardiomyocytes from NF‐κB Luciferase reporter mice. Taken together, our finding indicates that LPS acts through TLR4 and mediates a proinflammatory response in primary cardiomyocytes, in particular by production of IL‐6.

IL‐6 is a prominent proinflammatory cytokine, found to be increased in patients after acute coronary syndrome. Elevated IL‐6 levels are associated with increased infarct size, that is, indicating increased tissue damage and adverse outcomes [32]. After its release, IL‐6 can lead to myocardial dysfunction [33], which is not uncommon in septic patients [34]. In experimental rat studies, intraperitoneal injection of LPS causes cardiac dysfunction and damage [35]. Therefore, blocking IL‐6 release and its upstream activation pathways may be a potential strategy for preventing myocardial damage and dysfunction during sepsis.

Intraperitoneal LPS injection in mice increases serum levels of proinflammatory cytokines and myocardial mRNA expression of *IL‐6*, *TNF*, *IL‐1β*, and *MCP‐1* [36, 37] and a reduction in *TNF* release has been found after TLR4 inhibition [19]. We observed an increase of *IL‐1β* mRNA expression in perfused mouse hearts treated with LPS. In isolated cardiomyocytes, the increase in *IL‐1β, MCP‐1*, and *CXCL2* mRNA expression was reduced when blocking TLR4, indicating a cardiac‐specific response for these cytokines to LPS. Intraperitoneal LPS injection increased p‐JNK signaling in the myocardium up to 24 h postinjection, reducing phosphorylation by TLR4 inhibition (CLI‐095) [19]. Possibly systemic LPS exposure causes different upstream signaling activation pathways than in the isolated heart.

NF‐κB p65 phosphorylation increased in the isolated perfused heart after LPS administration. NF‐κB activation may cause production of several cytokines and chemokines, including IL‐6, CXCL2, IL‐1β, TNF, and MCP‐1 [38], corresponding to our findings of increased cytokines. This indicates that NF‐κB activation is involved in intracellular signaling in the isolated heart after LPS exposure and subsequent increased mRNA expression and secretion of cytokines.

Nonimmune cells, such as cardiomyocytes, express innate immune receptors [8, 10]. Previous studies showed no significant increase in cytokine production (IL‐1β, TNF, or IL‐6) in isolated cardiomyocytes from rats exposed to LPS for 1‐24 h [39]. After systemic *Klebsiella pneumoniae* administration, isolated rat cardiomyocytes had continued production of these cytokines 18 h after isolation [40]. In isolated neonatal rat cardiomyocytes, LPS exposure increased IL‐1β, TNF, and IL‐6 release [41]. TLR4 inhibition reduces the release of inflammatory cytokines and mortality during sepsis in mice [42]. In mice and rats, systemic TLR4 inhibition reduces TNF release and myocardial injury while improving cardiac function [19].

### 4.3 Damage Induced DAMP Release, an IR Model

We used IR in isolated hearts to investigate an isolated endogenous response to tissue damage and subsequent DAMP response without circulating immune cells and ongoing systemic inflammation. TLR4 inhibition significantly reduced infarct size, LDH release and IL‐6 release. These findings suggest a role of TLR4 in mediating tissue injury and inflammatory signaling during IRI. The inflammatory response to DAMPs are comparable to the LPS doses used in our study, indicating the severe effect of sterile inflammation after myocardial infarction. In a previous study, IL‐6 deficient mice had reduced infarct size in a model of myocardial IR in vivo [43]. This further implicates IL‐6 as an important cytokine during cardiac tissue damage. During sepsis, tissue damage releases DAMPs which enhance the inflammatory response, and subsequently increase tissue injury [44].

Our results indicate that TLR4 activation is both detrimental to cardiac cells and is part of the IL‐6‐dependent immune cell recruitment. To our knowledge, our study shows for the first time that inhibiting TLR4 at reperfusion reduced infarct size after global ischemia in isolated, perfused mouse hearts. Our results do not allow for speculation of which specific ligand is responsible for this detrimental effect. One suggested endogenous TLR4 ligand is high mobility group box 1 protein (HMGB1), which is known to activate the immune system, causing inflammation and organ damage [45]. LPS has been shown to upregulate HMGB1 in rat cardiomyocytes, resulting in negative inotropic effects [46], and HMGB1 is implicated in facilitating LPS entering cells, and subsequently triggering cell death during sepsis [47].

Blocking TLR4 at reperfusion decreased phosphorylation of ERK 1/2 compared to control. ERK 1/2 is a kinase that is involved in the MAPK pathway and regulates different cell processes, including inflammation, cell survival, and cell growth [21]. Activation of NF‐κB p65 did not change after TLR4 inhibition. Neither did expression of TLR4 related signaling genes. However, this assessment was conducted after 60 min of reperfusion, a timeframe that might be considered late for capturing these mechanistic changes.

### 4.4 DAMPs Versus PAMPs

Both PAMPs and DAMPs can be detrimental to the heart and cardiomyocytes during sepsis [48]. Our results revealed that exposure to infectious and sterile stimuli both led to a rise of proinflammatory cytokines such as IL‐6 (Supporting Information S1: Figure 5). LPS and IR exposure caused comparable IL‐6 release, highlighting the proinflammatory potential of DAMPs and PAMPs in driving inflammation.

This study aimed to address differences between PAMPs and DAMPs in signal transduction leading to inflammation. In mice, DAMPs produce a weaker immune response, less TLR‐signal desensitization, and contribute less to innate immune cell death [49]. When necrotic fibroblast supernatants was used as DAMPs and injected into mice intravenously, DAMPs caused higher mortality rate compared to PAMPs [49]. Eppensteiner et al. also discovered that multiple systemic injections of low pro‐coagulative DAMPs (supernatant from sonicated mouse pancreatic cancer cell line) in mice gradually increased proinflammatory cytokines and organ damage markers [49]. Mitochondrial DAMPs have a high proinflammatory role, probably due to its bacterial origin and high resemblance to bacterial PAMPs. More details on the role of Mitochondrial DAMPs in the heart has recently been reviewed [50, 51].

### 4.5 Limitation of the Study

In a clinical setting, sepsis and sterile inflammation are extremely complex due to complicated molecular signaling events in the tissue. Consequently, interpretations of findings from isolated organs and cells, lacking the influence of circulating immune cells, cannot be directly extrapolated to in vivo conditions. On the other hand, a reductionistic approach increase our understanding of isolated events in specific tissues. Very little is known about the direct effect of LPS on cardiomyocytes, and detailed information about such responses is necessary to understand the organs molecular response to sepsis.

Although we measured cytokines released continuously, we only collected tissue at the end of heart perfusion. Molecular responses could occur at an earlier time point. In particular, this might apply to the NF‐κB/p38 MAPK response, indicating that we have missed the early response but find the effect.

Working with cardiac tissue poses challenges, particularly due to its high protein density. Obtaining satisfactory protein yields in molecular assays, such as western blot, is difficult, given the low abundance of target protein relative to *e.g*. cardiac cytoskeleton. This may offer an explanation for our inability to detect phosphorylation of proteins in isolated perfused hearts. Additionally, changes in phosphorylation might have occurred earlier during reperfusion.

## 5 Conclusion

Perfusing isolated mouse hearts with either *E. coli* or LPS induced a large cardiac‐specific increase in cytokine expression and release, but only the LPS‐induced IL‐6 production was blocked by inhibiting TLR4. Following IRI, TLR4 blockade also resulted in a reduction of IL‐6 release. Accordingly, both PAMPs and DAMPs induced a cardiac‐specific inflammatory response through TLR4 with expression and release of IL‐6 mitigated by TLR4 inhibition.

Taken together, our findings suggest that cardiomyocytes, when exposed to pathogens ex vivo or in vitro, exhibit heightened activity in intracellular pathways in response to proinflammatory stimuli. This leads to a subsequent increase in mRNA expression and release of proinflammatory cytokines. Consequently, cardiomyocytes may play essential roles in the intricate immune response observed during sepsis.

## Author Contributions

**Christine W. Wiger:** conceptualization, data curation, formal analysis, methodology, roles/writing–original draft, writing–review and editing. **Trine Ranheim:** data curation, formal analysis, methodology, writing–review and editing. **Henriette Arnesen:** data curation, methodology, methodology, writing–review and editing. **Jarle Vaage:** formal analysis, supervision, writing–review and editing. **Søren E. Pischke:** data curation, methodology, project administration, writing–review and editing. **Arne Yndestad:** formal analysis, methodology, project administration, supervision; writing–review and editing. **Kåre‐Olav Stensløkken:** conceptualization, formal analysis, funding acquisition, methodology, project administration, supervision, writing–review and editing. **May‐Kristin Torp:** conceptualization, data curation, formal analysis, methodology, project administration, supervision, roles/writing–original draft, writing–review and editing.

## Conflicts of Interest

The authors declare no conflicts of interest.

## Supporting information

Supporting information.

## References

[1] M. Singer , C. S. Deutschman , C. W. Seymour , et al., “The Third International Consensus Definitions for Sepsis and Septic Shock (Sepsis‐3),” Journal of the American Medical Association 315 (2016): 801–810. 26903338 10.1001/jama.2016.0287 PMC4968574

[2] K. E. Rudd , S. C. Johnson , K. M. Agesa , et al., “Global, Regional, and National Sepsis Incidence and Mortality, 1990‐2017: Analysis for the Global Burden of Disease Study,” Lancet 395 (2020): 200–211. 31954465 10.1016/S0140-6736(19)32989-7 PMC6970225

[3] L. B. Kosyakovsky , F. Angriman , E. Katz , et al., “Association Between Sepsis Survivorship and Long‐Term Cardiovascular Outcomes in Adults: A Systematic Review and Meta‐Analysis,” Intensive Care Medicine 47 (2021): 931–942. 34373953 10.1007/s00134-021-06479-y

[4] X. Zhang , H. Liu , K. Hashimoto , S. Yuan , and J. Zhang , “The Gut‐Liver Axis in Sepsis: Interaction Mechanisms and Therapeutic Potential,” Critical Care 26 (2022): 213. 35831877 10.1186/s13054-022-04090-1 PMC9277879

[5] D. V. Krysko , P. Agostinis , O. Krysko , et al., “Emerging Role of Damage‐Associated Molecular Patterns Derived From Mitochondria in Inflammation,” Trends in Immunology 32 (2011): 157–164. 21334975 10.1016/j.it.2011.01.005

[6] O. Takeuchi and S. Akira , “Pattern Recognition Receptors and Inflammation,” Cell 140 (2010): 805–820. 20303872 10.1016/j.cell.2010.01.022

[7] A. S. Sameer and S. Nissar , “Toll‐Like Receptors (TLRs): Structure, Functions, Signaling, and Role of Their Polymorphisms in Colorectal Cancer Susceptibility,” BioMed Research International 2021 (2021): 1157023. 34552981 10.1155/2021/1157023 PMC8452412

[8] J. A. Mitchell , B. Ryffel , V. F. J. Quesniaux , N. Cartwright , and M. Paul‐Clark , “Role of Pattern‐Recognition Receptors in Cardiovascular Health and Disease,” Biochemical Society Transactions 35 (2007): 1449–1452. 18031243 10.1042/BST0351449

[9] M. Nishimura and S. Naito , “Tissue‐Specific Mrna Expression Profiles of Human Toll‐Like Receptors and Related Genes,” Biological and Pharmaceutical Bulletin 28 (2005): 886–892. 15863899 10.1248/bpb.28.886

[10] L. Lin and A. A. Knowlton , “Innate Immunity and Cardiomyocytes in Ischemic Heart Disease,” Life Sciences 100 (2014): 1–8. 24486305 10.1016/j.lfs.2014.01.062 PMC3970925

[11] Y. Umemura , H. Ogura , K. Takuma , et al., “Current Spectrum of Causative Pathogens in Sepsis: A Prospective Nationwide Cohort Study in Japan,” International Journal of Infectious Diseases 103 (2021): 343–351. 33221519 10.1016/j.ijid.2020.11.168

[12] D. Mokady , U. Gophna , and E. Z. Ron , “Virulence Factors of Septicemic *Escherichia coli* Strains,” International Journal of Medical Microbiology 295 (2005): 455–462. 16238019 10.1016/j.ijmm.2005.07.007

[13] S. M. Dauphinee and A. Karsan , “Lipopolysaccharide Signaling in Endothelial Cells,” Laboratory Investigation 86 (2006): 9–22. 16357866 10.1038/labinvest.3700366

[14] J. N. Fullerton , E. Segre , R. P. De Maeyer , A. A. Maini , and D. W. Gilroy , “Intravenous Endotoxin Challenge in Healthy Humans: An Experimental Platform to Investigate and Modulate Systemic Inflammation,” Journal of Visualized Experiments, no. 111 (2016): 53913, https://pubmed.ncbi.nlm.nih.gov/27213711/. 27213711 10.3791/53913 PMC4942172

[15] S. J. Burke , D. Lu , T. E. Sparer , et al., “NF‐κB and STAT1 Control CXCL1 and CXCL2 Gene Transcription,” American Journal of Physiology‐Endocrinology and Metabolism 306 (2014): E131–E149. 24280128 10.1152/ajpendo.00347.2013 PMC3920007

[16] T. Kawai and S. Akira , “Signaling to NF‐κB by Toll‐Like Receptors,” Trends in Molecular Medicine 13 (2007): 460–469. 18029230 10.1016/j.molmed.2007.09.002

[17] Y. Feng , L. Zou , M. Zhang , Y. Li , C. Chen , and W. Chao , “MyD88 and Trif Signaling Play Distinct Roles in Cardiac Dysfunction and Mortality During Endotoxin Shock and Polymicrobial Sepsis,” Anesthesiology 115 (2011): 555–567. 21792053 10.1097/ALN.0b013e31822a22f7 PMC3162066

[18] J. Oyama , C. Blais, Jr. , X. Liu , et al., “Reduced Myocardial Ischemia‐Reperfusion Injury in Toll‐Like Receptor 4‐Deficient Mice,” Circulation 109 (2004): 784–789. 14970116 10.1161/01.CIR.0000112575.66565.84

[19] C. Chang , L. Hu , S. Sun , et al., “Regulatory Role of the TLR4/JNK Signaling Pathway in Sepsis‐Induced Myocardial Dysfunction,” Molecular Medicine Reports 23 (2021): 334. 33760172 10.3892/mmr.2021.11973 PMC7974310

[20] J. C. Alves‐Filho , A. de Freitas , M. Russo , and F. Q. Cunha , “Toll‐Like Receptor 4 Signaling Leads to Neutrophil Migration Impairment in Polymicrobial Sepsis,” Critical Care Medicine 34 (2006): 461–470. 16424729 10.1097/01.ccm.0000198527.71819.e1

[21] K. O. Stensløkken , A. Rutkovskiy , M. L. Kaljusto , A. D. Hafstad , T. S. Larsen , and J. Vaage , “Inadvertent Phosphorylation of Survival Kinases in Isolated Perfused Hearts: A Word of Caution,” Basic Research in Cardiology 104 (2009): 412–423. 19198917 10.1007/s00395-009-0780-1

[22] E. B. Thorgersen , S. E. Pischke , A. Barratt‐Due , et al., “Systemic CD14 Inhibition Attenuates Organ Inflammation in Porcine *Escherichia coli* Sepsis,” Infection and Immunity 81 (2013): 3173–3181. 23774598 10.1128/IAI.00390-13 PMC3754210

[23] T. D. O'connell , M. C. Rodrigo , and P. C. Simpson , “Isolation and Culture of Adult Mouse Cardiac Myocytes,” Methods in Molecular Biology 357 (2007): 271–296. 17172694 10.1385/1-59745-214-9:271

[24] H. Carlsen , J. Ø. Moskaug , S. H. Fromm , and R. Blomhoff , “In Vivo Imaging of NF‐Kappa B Activity,” Journal of Immunology 168 (2002): 1441–1446. 10.4049/jimmunol.168.3.1441 11801687

[25] T. Peng , “Inhibition of p38 MAPK Decreases Myocardial TNF‐Alpha Expression and Improves Myocardial Function and Survival in Endotoxemia,” Cardiovascular Research 59 (2003): 893–900. 14553829 10.1016/s0008-6363(03)00509-1

[26] G. Zanetti , D. Heumann , J. Gérain , et al., “Cytokine Production After Intravenous or Peritoneal Gram‐Negative Bacterial Challenge in Mice. Comparative Protective Efficacy of Antibodies to Tumor Necrosis Factor‐Alpha and to Lipopolysaccharide,” Journal of Immunology 148 (1992): 1890–1897. 1541827

[27] N. Long , J. Deng , M. Qiu , et al., “Inflammatory and Pathological Changes in *Escherichia coli* Infected Mice,” Heliyon 8 (2022): e12533. 36643320 10.1016/j.heliyon.2022.e12533 PMC9834738

[28] L. Oliveira‐Nascimento , P. Massari , and L. M. Wetzler , “The Role of TLR2 in Infection and Immunity,” Frontiers in Immunology 3 (2012): 79. 22566960 10.3389/fimmu.2012.00079 PMC3342043

[29] A. Dalpke , J. Frank , M. Peter , and K. Heeg , “Activation of Toll‐Like Receptor 9 by DNA From Different Bacterial Species,” Infection and Immunity 74 (2006): 940–946. 16428738 10.1128/IAI.74.2.940-946.2006 PMC1360326

[30] C. Alter , A. S. Henseler , C. Owenier , et al., “IL‐6 in the Infarcted Heart Is Preferentially Formed by Fibroblasts and Modulated By Purinergic Signaling,” Journal of Clinical Investigation 133 (2023): e163799. 36943408 10.1172/JCI163799 PMC10232006

[31] S. Yang , R. Zheng , S. Hu , et al., “Mechanism of Cardiac Depression after Trauma‐Hemorrhage: Increased Cardiomyocyte IL‐6 and Effect of Sex Steroids on IL‐6 Regulation and Cardiac Function,” American Journal of Physiology‐Heart and Circulatory Physiology 287 (2004): H2183–H2191. 15475534 10.1152/ajpheart.00624.2003

[32] C. Tiller , M. Reindl , M. Holzknecht , et al., “Association of Plasma interleukin‐6 with Infarct Size, Reperfusion Injury, and Adverse Remodelling After ST‐Elevation Myocardial Infarction,” European Heart Journal. Acute Cardiovascular Care 11 (2022): 113–123. 34849677 10.1093/ehjacc/zuab110

[33] T. Jiang , D. Peng , W. Shi , et al, “IL‐6/STAT3 Signaling Promotes Cardiac Dysfunction by Upregulating FUNDC1‐Dependent Mitochondria‐Associated Endoplasmic Reticulum Membranes Formation in Sepsis Mice,” Frontiers in Cardiovascular Medicine 8 (2021): 790612. 35118141 10.3389/fcvm.2021.790612 PMC8804492

[34] K. R. Walley , “Sepsis‐Induced Myocardial Dysfunction,” Current Opinion in Critical Care 24 (2018): 292–299. 29846206 10.1097/MCC.0000000000000507

[35] Z. Wang , L. Bu , P. Yang , S. Feng , and F. Xu , “Alleviation of Sepsis‐Induced Cardiac Dysfunction by Overexpression of Sestrin2 Is Associated With Inhibition of p‐S6K and Activation of the p‐AMPK Pathway,” Molecular Medicine Reports 20 (2019): 2511–2518. 31524263 10.3892/mmr.2019.10520 PMC6691248

[36] N. Li , H. Zhou , H. Wu , et al., “STING‐IRF3 Contributes to Lipopolysaccharide‐Induced Cardiac Dysfunction, Inflammation, Apoptosis and Pyroptosis by Activating NLRP3,” Redox Biology 24 (2019): 101215. 31121492 10.1016/j.redox.2019.101215 PMC6529775

[37] L. Xianchu , Z. Lan , L. Ming , and M. Yanzhi , “Protective Effects of Rutin on Lipopolysaccharide‐Induced Heart Injury in Mice,” Journal of Toxicological Sciences 43 (2018): 329–337. 29743444 10.2131/jts.43.329

[38] T. Liu , L. Zhang , D. Joo , and S. C. Sun , “NF‐κB Signaling in Inflammation,” Signal Transduction and Targeted Therapy 2 (2017): 17023. 29158945 10.1038/sigtrans.2017.23 PMC5661633

[39] A. D. Niederbichler , M. V. Westfall , G. L. Su , et al., “Cardiomyocyte Function After Burn Injury and Lipopolysaccharide Exposure: Single‐Cell Contraction Analysis and Cytokine Secretion Profile,” Shock 25 (2006): 176–183. 16525357 10.1097/01.shk.0000192123.91166.e1

[40] J. W. Horton , “A Model of Myocardial Inflammation and Dysfunction in Burn Complicated by Sepsis,” Shock 28 (2007): 326–333. 17529909 10.1097/01.shk.0000238064.54332.c8

[41] Y. Qiao , L. Wang , T. Hu , D. Yin , H. He , and M. He , “Capsaicin Protects Cardiomyocytes Against Lipopolysaccharide‐Induced Damage via 14‐3‐3γ‐Mediated Autophagy Augmentation,” Frontiers in Pharmacology 12 (2021): 659015. 33986684 10.3389/fphar.2021.659015 PMC8111444

[42] T. Sha , M. Sunamoto , T. Kitazaki , J. Sato , M. Ii , and Y. Iizawa , “Therapeutic Effects of TAK‐242, a Novel Selective Toll‐Like Receptor 4 Signal Transduction Inhibitor, in Mouse Endotoxin Shock Model,” European Journal of Pharmacology 571 (2007): 231–239. 17632100 10.1016/j.ejphar.2007.06.027

[43] W. M. C. Jong , H. Ten Cate , A. C. Linnenbank , et al., “Reduced Acute Myocardial Ischemia‐Reperfusion Injury in IL‐6‐Deficient Mice Employing a Closed‐Chest Model,” Inflammation Research 65 (2016): 489–499. 26935770 10.1007/s00011-016-0931-4 PMC4841857

[44] N. L. Denning , M. Aziz , S. D. Gurien , and P. Wang , “DAMPs and NETs in Sepsis,” Frontiers in Immunology 10 (2019): 2536. 31736963 10.3389/fimmu.2019.02536 PMC6831555

[45] A. Murao , M. Aziz , H. Wang , M. Brenner , and P. Wang , “Release Mechanisms of Major Damps,” Apoptosis 26 (2021): 152–162. 33713214 10.1007/s10495-021-01663-3 PMC8016797

[46] S. Hagiwara , H. Iwasaka , T. Uchino , and T. Noguchi , “High Mobility Group Box 1 Induces a Negative Inotropic Effect on the Left Ventricle in an Isolated Rat Heart Model of Septic Shock: A Pilot Study,” Circulation Journal 72 (2008): 1012–1017. 18503231 10.1253/circj.72.1012

[47] M. Deng , Y. Tang , W. Li , et al., “The Endotoxin Delivery Protein HMGB1 Mediates Caspase‐11‐Dependent Lethality in Sepsis,” Immunity 49 (2018): 740–753.e7. 30314759 10.1016/j.immuni.2018.08.016 PMC6300139

[48] R. Habimana , I. Choi , H. J. Cho , D. Kim , K. Lee , and I. Jeong , “Sepsis‐Induced Cardiac Dysfunction: A Review of Pathophysiology,” Acute and Critical Care 35 (2020): 57–66. 32506871 10.4266/acc.2020.00248 PMC7280799

[49] J. Eppensteiner , J. Kwun , U. Scheuermann , et al., “Damage‐ and Pathogen‐Associated Molecular Patterns Play Differential Roles in Late Mortality after Critical Illness,” JCI Insight 4 (2019): e127925. 31434802 10.1172/jci.insight.127925 PMC6777836

[50] M. K. Torp , K. O. Stensløkken , and J. Vaage , “When Our Best Friend Becomes Our Worst Enemy: The Mitochondrion in Trauma, Surgery, and Critical Illness,” Journal of Intensive Care Medicine, (2024): 8850666241237715. 10.1177/08850666241237715 38505947

[51] M. K. Torp , J. Vaage , and K. O. Stensløkken , “Mitochondria‐Derived Damage‐Associated Molecular Patterns and Inflammation in the Ischemic‐Reperfused Heart,” Acta Physiol 237 (2023): e13920. 10.1111/apha.13920 36617670

