# Supplementary material for: TLR4 Inhibition Attenuated LPS‐Induced Proinflammatory Signaling and Cytokine Release in Mouse Hearts and Cardiomyocytes
Source: Immun Inflamm Dis. 2025 Jan 24;13(1):e70133. doi: 10.1002/iid3.70133 (PMC11760985; doi:10.1002/iid3.70133)
Supplement: Supplementary file 1 — Supporting information. [file IID3-13-e70133-s001.docx]

**Supplementary data**

**Supplementary figure 1**

**
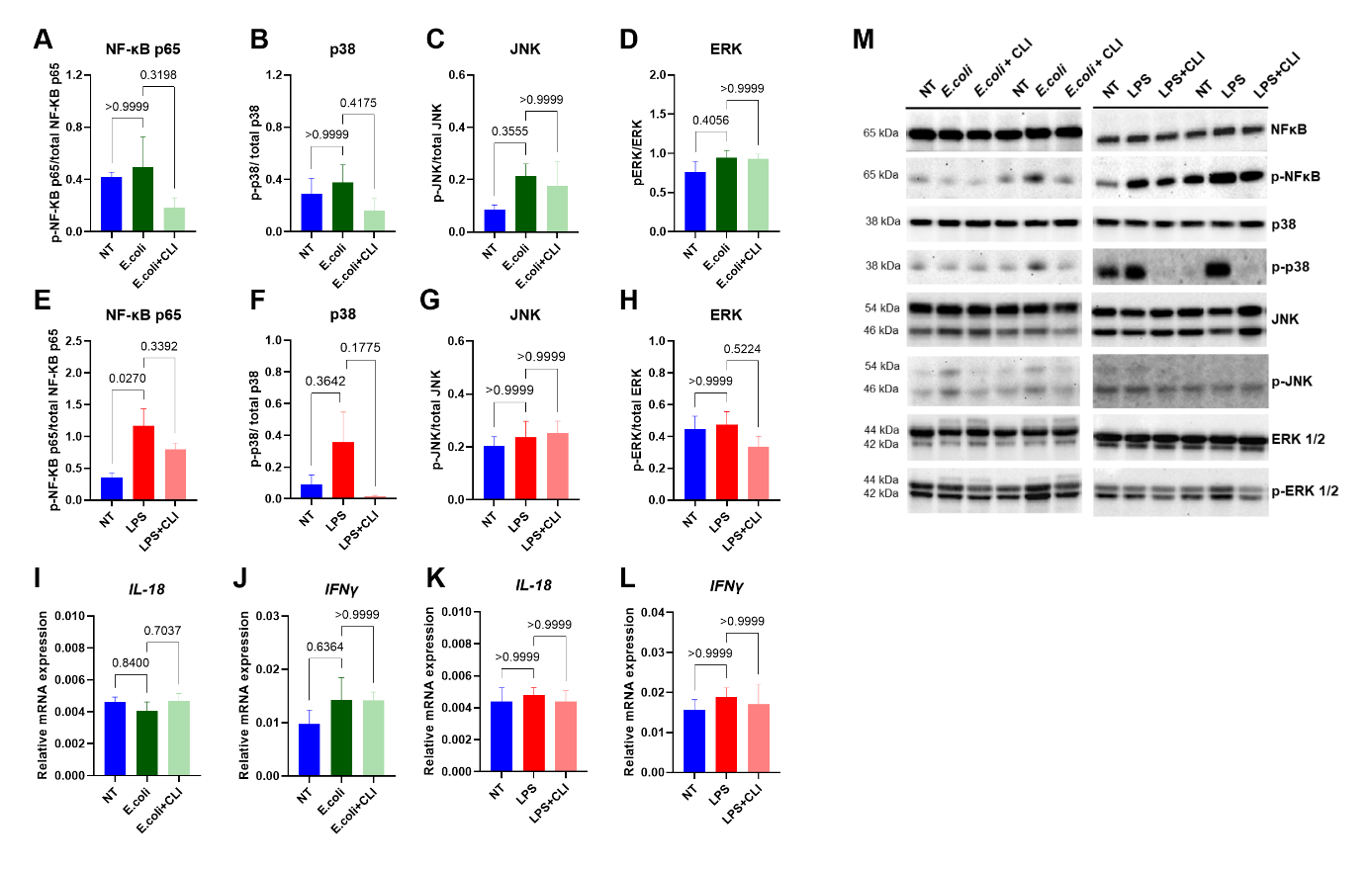
**

**Figure 1: PAMPs activated pro-inflammatory signaling in the isolated perfused heart**

Isolated, buffer-perfused hearts (n=5-6) were introduced to either (i) no treatment (NT; blue), (ii) *Escherichia coli* (*E.coli*; dark green), (iii) *E.coli* and CLI-095 (*E.coli*+CLI; light green), (iv) lipopolysaccharide (LPS; red), or (v) LPS and CLI-095 (LPS+CLI; light red) for 120 minutes. Activation of NF-κB p65, p38, JNK, and ERK were investigated in tissue lysates by western blotting and is presented as a ratio between phosphorylated (p) and total protein.

**Supplementary figure 2**
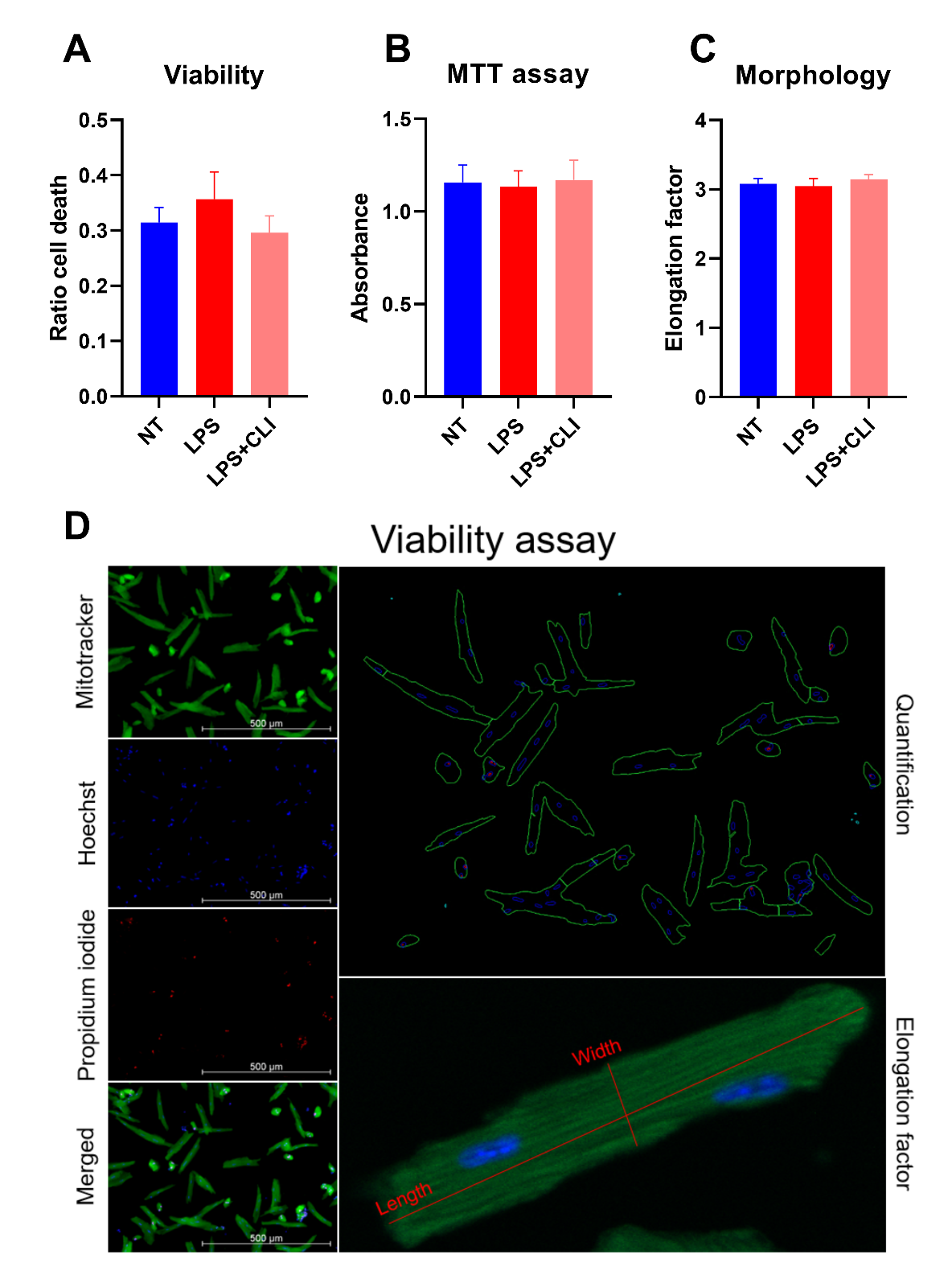


**Supplementary Figure 2: Cardiomyocyte viability and morphology with LPS treatment**

Cardiomyocytes were exposed to (i) no treatment (NT; blue), (ii) lipopolysaccharide (LPS; red), or (iii) LPS and CLI-095 (LPS+CLI; light red). Cardiomyocyte viability was investigated with (A) high-throughput microscopy quantifying cell death with a ratio of the number of propidium iodide stained nuclei and the total number of Hoechst stained nuclei (illustrated in panel D) (n=12), and (B) MTT assay (n=8). Cardiomyocytes morphology was investigated by measuring the elongation factor (ratio of cardiomyocyte width and length as illustrated in panel D, n=8). Data are presented as mean±SEM and analyzed with one-way ANOVA and Bonferroni's multiple comparisons.

**Supplementary figure 3**


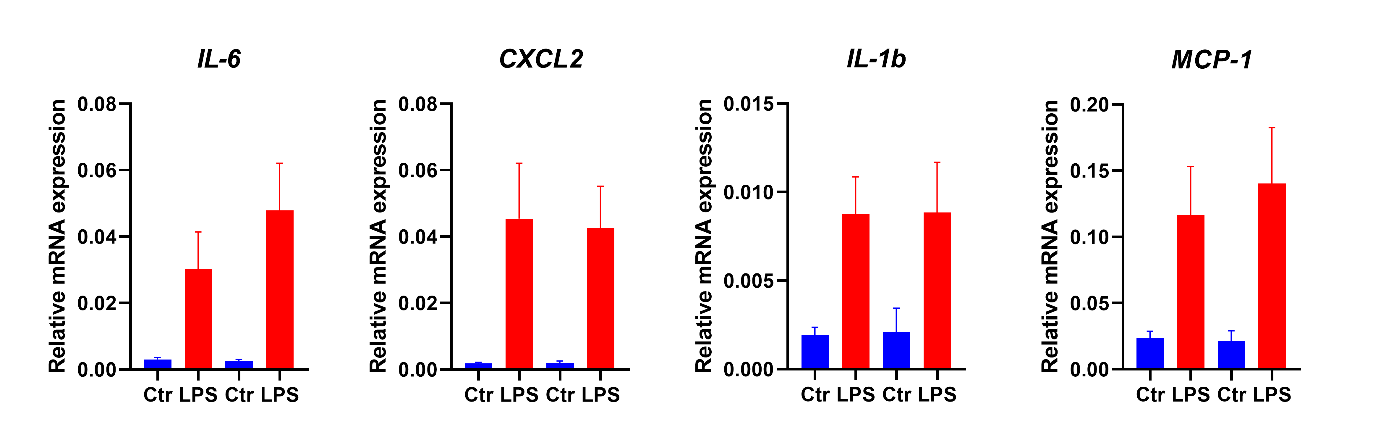


**Supplementary figure 3:**

mRNA expression of pro-inflammatory cytokines investigated in primary adult cardiomyocytes (n=5) treated with (i) no treatment (NT; blue) or (ii) lipopolysaccharide (LPS; red) and exposed to normoxia (solid) or hypoxia-reoxygenation (H/R, gridlines). Data are presented as mean±SEM, and analyzed with Kruskal Wallace and Dunn’s multiple comparisons test (A, C, and D) or one-way ANOVA and Šidák’s multiple comparison test (B).

Abbreviations: NF-κB (Nuclear factor kappa B), MAPK (mitogen-activated protein kinase), IL-6 (interleukin 6), CXCL2 (C-X-C Motif Chemokine Ligand 2), IL-1β (interleukin 1 beta).

**Supplementary figure 4**


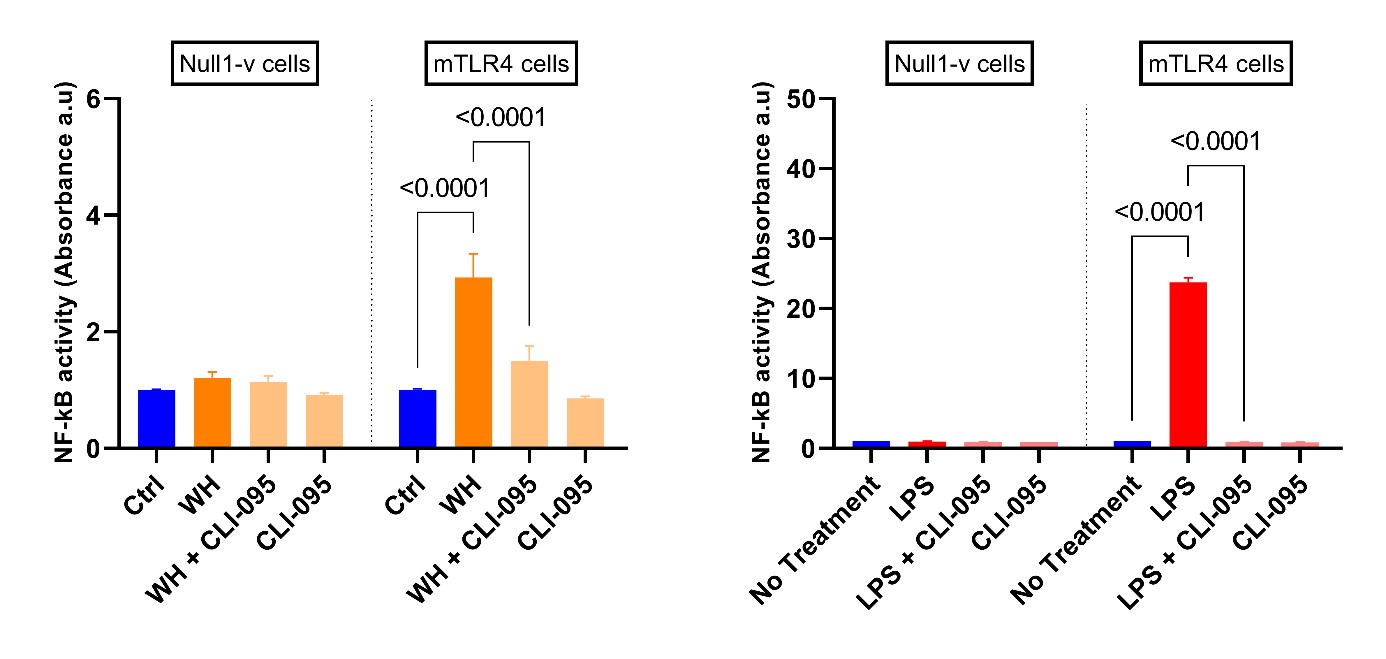


**Supplementary figure 4:**

mTLR4 overexpressed HEK cells and the control Null cell line exposed to (A), n=3 (i) no treatment (NT; blue), (ii) lipopolysaccharide (LPS; red), (iii) LPS and CLI-095 (LPS+CLI; light red), and (iv) CLI-095 (CLI: light pink), or (B) n=12 (i) no treatment (NT; blue), (ii) whole heart cellular debris (WH; orange), (iii) WH and CLI-095 (WH+CLI; light orange), or (iv) CLI-+095 (CLI; light orange). Data are presented as mean±SEM and analyzed with one-way ANOVA (A and B), and Bonferroni's multiple comparisons.

Abbreviations: NF-κB (Nuclear factor kappa B), MAPK (mitogen-activated protein kinase), IL-6 (interleukin 6), CXCL2 (C-X-C Motif Chemokine Ligand 2), IL-1β (interleukin 1 beta).

**Supplementary figure 5**


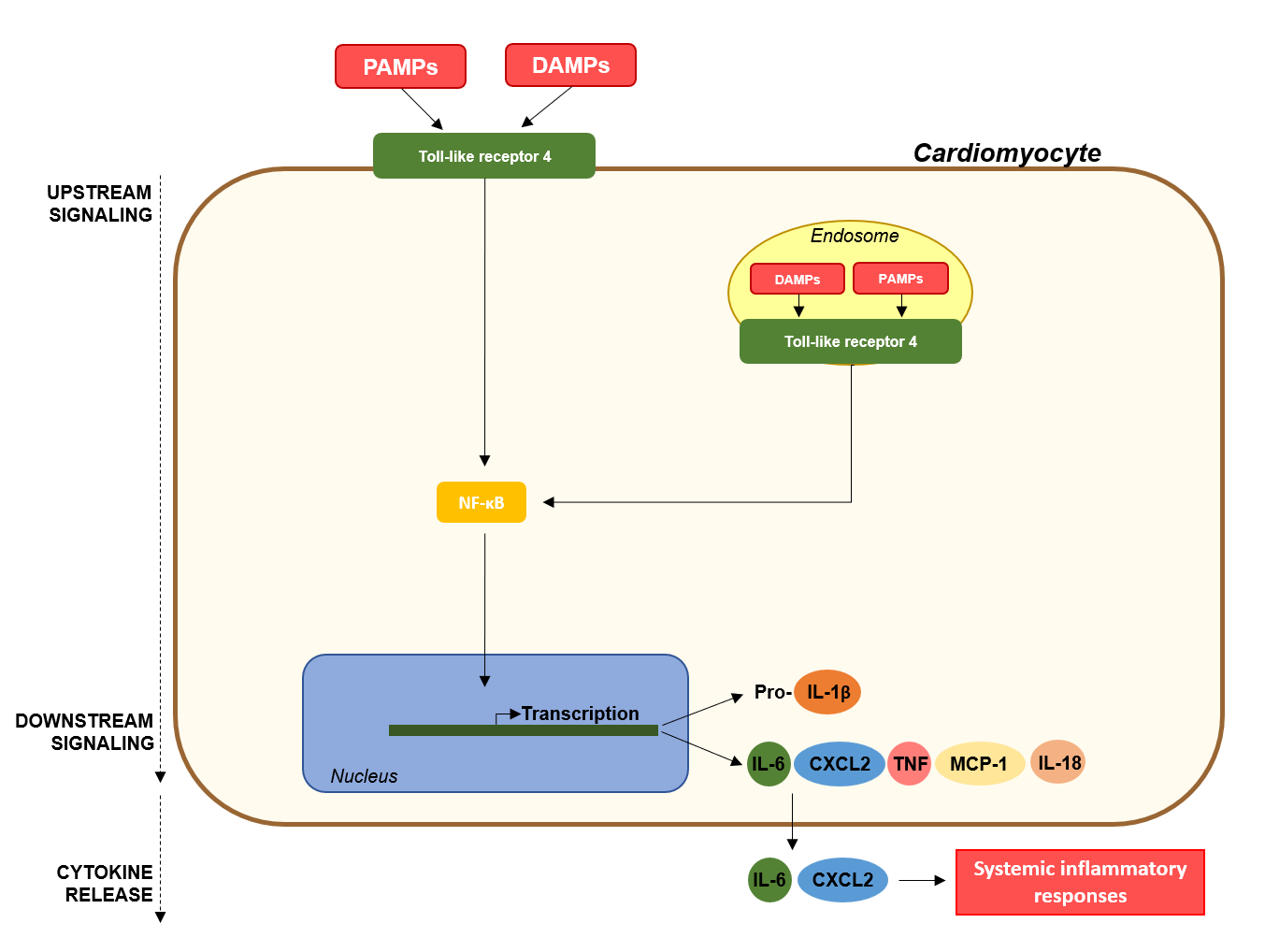


**Supplementary figure 5: Schematic overview of PAMPs and DAMPs activation of pro-inflammatory signaling pathways and cytokine release in primary adult cardiomyocytes**

In primary adult cardiomyocytes, PAMPs (*Escherichia coli* and lipopolysaccharide) or DAMPs (released through ischemia-reperfusion injury) binds to toll-like receptor 4 and activates pro-inflammatory signaling through the NF-κB. This trigger expression of cytokines *(e.g.* IL-.6, CXCL2, and pro-IL-1β)*,* which subsequently are release across the sarcolemma and into the extracellular milieu resulting in systemic inflammatory responses.

Abbreviations: NF-κB (Nuclear factor kappa B), MAPK (mitogen-activated protein kinase), IL-6 (interleukin 6), CXCL2 (C-X-C Motif Chemokine Ligand 2), IL-1β (interleukin 1 beta).

**Supplementary table 1: Antibodies for western blotting**

| **Manufacturer** | **Antibody** | **Catalogue number** | **kDa** | **Dilution** | **Dilution reagent** |
| --- | --- | --- | --- | --- | --- |
| CellSignaling | NF-KB | 8242S | 65kDa | 1:1.000 | 5% BSA |
| CellSignaling | Phospho NF-KB | 3033S | 65kDa | 1:1.000 | 5% BSA |
| CellSignaling | ERK | 9102S | 42/44 kDa | 1:1.000 | 5% milk |
| CellSignaling | Phospho-ERK | 9101S | 42/44 kDa | 1:1.000 | 5% milk |
| CellSignaling | JNK | 9252S | 46/54 kDa | 1:1.000 | 5% milk |
| CellSignaling | Phospho-JNK | 9251S | 46/54 kDa | 1:1.000 | 5% milk |
| CellSignaling | p38 | 9212S | 38 kDa | 1:1.000 | 5% milk |
| CellSignaling | Phospho-p38 | 9211S | 38 kDa | 1:1.000 | 5% milk |
| Southern BioTech | Goat-anti-Rabbit | 4030-05 | N/A | 1:20.000 | 5% milk |

**Supplementary table 2: Primer sequences**

| Gene name | Forward primer (5’-3’) | Reverse primer (5’-3’) |
| --- | --- | --- |
| *IL-6* | TCTAATTCATATCTTCAACCAAGAGGTAA | GAATTGGATGGTCTTGGTCCTTA |
| *CXCL2* | AGTGAACTGCGCTGTCAATG | CAGGGTCAAGGCAAACTTTTT |
| *IL-1β* | TGAAATGCCACCTTTTGACA | TGTCCTCATCCTGGAAGGTC |
| *TNF* | GAACTGGCAGAAGAGGCACT | GGTCTGGGCCATAGAACTGA |
| *MCP-1* | CCCAATGAGTAGGCTGGAGA | GCTGAAGACCTTAGGGCAGA |
| *IL-18* | GGCTGCCATGTCAGAAGACT | GGGTTCACTGGCACTTTGAT |
| *IFNγ* | ATTGCCAAGTTTGAGGTCAACA | CGCTTCCTGAGGCTGGATT |
| *Rpl32* | TCGTCAAAAAGAGGACCAAGAAG | CCGCCAGTTTCGCTTAATTT |

**Supplementary methods**

*Hypoxia-reoxygenation (H/R) of primary adult cardiomocytes*

One day prior to the H/R experiments, hypoxic medium (Special MEM [-] glucose (Ref. no. 074-1183P), Gibco, Thermo Fisher Scientific), supplemented with 4.2 mM NaHCO_3_, 100 U/mL penicillin-streptomycin, 2 mM L-glutamine, 0.1% BSA (low endotoxin, fatty acid free), and 1 mM BDM) was placed in a hypoxic chamber (#856-HYPO, Plas Labs, Lansing, MI) to achieve 1% O_2_ and 2% CO_2_. Isolated cardiomyocytes were placed in the hypoxic chamber and washed once with hypoxic medium before cultured for 40 minutes at 37 ºC in 1% O_2_ and 2% CO_2_. The cells were then reoxygenated (21% O_2_ and 2% CO_2_) for 2 hours in normoxic medium (Special MEM [-] glucose supplemented with 4.2 mM NaHCO_3_, 100 U/mL penicillin-streptomycin, 2 mM L-glutamine, 0.1% BSA (low endotoxin, fatty acid free), 1 mM BDM, and 5 mM glucose). Control cells were kept in normoxic incubator with normoxic medium and received similar media changes as H/R treated cells.

**Murine TLR4 over-expressing HEK293 cells**

The commercial available HEK-Blue™ mTLR4 cell line (Invivogen), stably transfected with murine TLR4, MD-2 and CD14 co‑receptor genes, and an NF-κB-inducible SEAP (secreted embryonic alkaline phosphatase) reporter gene, was used to assess TLR4-dependent NF-κB activity. Their parental cell line HEK-Blue Null1-v (Invivogen) served as negative control. Both cell types were cultured according to the manufacturer’s instructions. Detection of NF-κB-induced SEAP production was determined with the use of HEK-Blue™ Detection medium (Invivogen) and carried out according to the manufacturer`s protocol. In short, 40.000 cells per well were seeded in a 96-well plate. NF-κB activity was measured with a BioTek PowerWave XS Microplate Spectrophotometer (BioTek Instruments Inc., Winooski, VT) at 630 nm, based on the accumulation of hydrolyzed SEAP color substrate in the medium.
